# AI-powered retinal imaging for early ASCVD risk stratification in a hybrid care model: preliminary findings from SAFER

**DOI:** 10.3389/frai.2026.1920911

**Published:** 2026-09-03

**Authors:** Hala Zakaria, Juman Ali, Mohammed Gouda Ibrahim, Idalys Roman, Ali Hashemi, Ihsan Almarzooqi

**Affiliations:** Metabolic, Dubai, United Arab Emirates

**Keywords:** cardiometabolic disease, cardiovascular risk, digital health, fundus photography, retinal image

## Abstract

**Background:**

AI-enabled retinal imaging provides a non-invasive method for assessing cardiovascular risk by identifying microvascular changes linked to atherosclerotic cardiovascular disease. This study assessed the feasibility and clinical associations of the AI-derived retinal risk score (Reti-CVD) in a hybrid care model and compared its categorical alignment with the American Heart Association PREVENT equation as a comparator risk model.

**Methods:**

In this retrospective cross-sectional study, 1,461 adults aged ≥40 at Metabolic underwent non-mydriatic retinal imaging. Fundus photographs were analyzed using the Dr. Noon AI platform to classify Reti-CVD risk. Associations with cardiometabolic characteristics were examined, and categorical agreement with PREVENT was assessed using Cohen’s kappa. ROC analyses evaluated the alignment of the continuous Reti-CVD score with PREVENT thresholds.

**Results:**

Higher Reti-CVD risk was significantly linked to older age, male sex, diabetes, hypertension, elevated HbA1c, higher blood pressure, lower HDL cholesterol, higher triglycerides, and older AI-predicted vascular age (all *p* < 0.001). After adjustment, age, female sex, and current smoking were independent predictors. The Reti-CVD score showed moderate discrimination for PREVENT-defined risk thresholds (AUC 0.689–0.757) and slight to fair agreement with PREVENT (*κ* = 0.144; weighted κ = 0.241). Among patients classified as low risk by PREVENT, 49.1% were categorized as moderate or high by Reti-CVD.

**Conclusion:**

AI-enabled retinal imaging was feasible in a hybrid setting and was associated with clinically plausible cardiometabolic risk gradients. Reti-CVD showed limited categorical agreement with PREVENT. However, the findings are hypothesis-generating and require prospective validation against ASCVD events before claims, improved prediction, or beneficial reclassification.

## 1 Introduction

Atherosclerotic cardiovascular disease (ASCVD) persists to be the leading cause of mortality and morbidity worldwide, with an estimated 17.9 million deaths annually, accounting for approximately one-third of all global deaths (Cardiovascular Diseases (CVDs), 2025). ASCVD encompasses a range of conditions, including coronary heart disease, peripheral artery disease, and aortic atherosclerotic disease. All of these conditions are a result of the progressive accumulation of atherosclerotic plaques within the arterial walls [American Heart Association (ASCVD), 2025]. However, these conditions often progress over decades before they develop into acute clinical events such as myocardial infarction or stroke (Mechanic et al., 2023; Tadi and Lui, 2023). As a result, there has been a shift toward developing risk estimation tools to enable early detection and accurate risk stratification.

To address this need, the development of several risk estimation tools has been adopted to predict ASCVD risk. Examples of these tools are the Framingham Risk Score, QRISK3, and the Pooled Cohort Equation, which have been used to predict the ASCVD risk at 30 and 10 years, respectively (Goff et al., 2014; Hippisley-Cox et al., 2017). It is important to note that these tools take traditional risk factors such as age, sex, blood pressure, lipid levels, diabetes status, and smoking history into consideration. Despite contributing to cardiovascular risk management, these tools are not without limitations. A trend was noticed across several studies that showed that they may underestimate the risk in younger populations and women, while also tending to overestimate the risk in the elderly (DeFilippis et al., 2017; Yeboah et al., 2012). Additionally, these tools fail to identify subclinical diseases, which interfere with early detection, hence not allowing for timely intervention (Talha et al., 2024).

The American Heart Association (AHA) unveiled the PREVENT (Predicting Risk of cardiovascular disease EVENTs) equation as a revised model for predicting cardiovascular risk, aimed at enhancing risk assessment by including additional clinical and metabolic factors, such as indicators of kidney function and cardiometabolic health (Khan et al., 2024; Razavi et al., 2024). Although PREVENT is a step forward compared to previous equations, it still fundamentally relies on standard clinical and laboratory data and does not directly evaluate subclinical vascular structure or microvascular damage.

With the increasing prevalence of cardiometabolic disorders, including diabetes, obesity, and dyslipidemia, all being major risk factors for vascular complications, and the extended asymptomatic phase of ASCVD, there is a growing need for the development of more sensitive, non-invasive, and scalable methods for risk assessment (Matheus et al., 2013; Vaduganathan et al., 2022) at the primary care level. The emergence of artificial intelligence (AI), especially through deep learning algorithms, has revolutionized medical imaging. AI enables automatic feature extraction, risk prediction, and diagnostic classification from imaging data. It has shown superior performance across various medical fields, including radiology, dermatology, and ophthalmology (De et al., 2020; Li et al., 2023; Mayo and Leung, 2018).

The use of AI in cardiac domains has been on the rise, where it has been applied to electrocardiograms, chest radiographs, and coronary CT angiograms to predict the risk of coronary episodes and improve diagnostic accuracy (Mayo Clinic, 2025; Bennani et al., 2023; Liao et al., 2022). Among these modalities, retinal imaging is considered the most promising non-invasive one. The retina offers a unique and accessible window into microvascular health, while AI models trained on fundus photographs have successfully predicted cardiovascular risk factors such as blood pressure, age, smoking status, and cardiac events (Poplin et al., 2018). The retinal AI (Reti-CVD) analysis can overcome many of the limitations associated with the traditional risk calculator by leveraging biomarkers obtained from images, hence offering a potential early detection leading to personalized treatment approaches. The use of AI retinal analysis to detect diabetic retinopathy is well established; however, its use to predict ASCVD risk remains in the early stages of clinical integration. Therefore, this creates a growing need to develop scalable, non-invasive, and feasible tools for ASCVD screening, especially in hybrid care settings that blend digital and in-clinic services.

This study aims to examine the relationship between Reti-CVD risk categories and established cardiometabolic risk factors to characterize feasibility, association patterns, and areas of discordance. It will also assess the categorical agreement and score-level alignment between the Reti-CVD and the PREVENT equation by the AHA.

## 2 Methodology

### 2.1 Study design and setting

SAFER (Screening for ASCVD using Fundus-based AI for Early Risk detection) is a single-center, retrospective cross-sectional observational implementation study conducted at Metabolic (formerly GluCare.Health), a technology-enabled center in Dubai, United Arab Emirates. Retinal imaging was performed as part of routine clinical care during scheduled outpatient follow-up or screening visits between November 2024 and January 2026. Because this was a cross-sectional retrospective analysis, no longitudinal ASCVD outcomes were available.

### 2.2 Participant selection

Patients were considered eligible for inclusion if they were adults aged 40 years or older, were attending Metabolic (formerly known as GluCare.health) for routine follow-up or screening, had at least one established cardiometabolic risk factor (type 2 diabetes mellitus, prediabetes, hypertension, obesity, dyslipidemia, or a family history of premature cardiovascular disease), and were able to undergo non-mydriatic retinal photography.

Patients were excluded if they had: a known history of established cardiovascular disease, defined as prior myocardial infarction, ischemic stroke, or coronary revascularization; retinal image quality insufficient for algorithmic analysis owing to media opacities such as dense cataracts; retinal pathologies unrelated to ASCVD risk, including retinal detachment; or current pregnancy. Patients for whom only one eye produced an ungradable image were retained in the analysis, as the AI algorithm is capable of generating a risk score from a single gradable fundus image. Patients for whom both eyes produced ungradable images were excluded.

### 2.3 Cohort derivation

**Figure 1:**
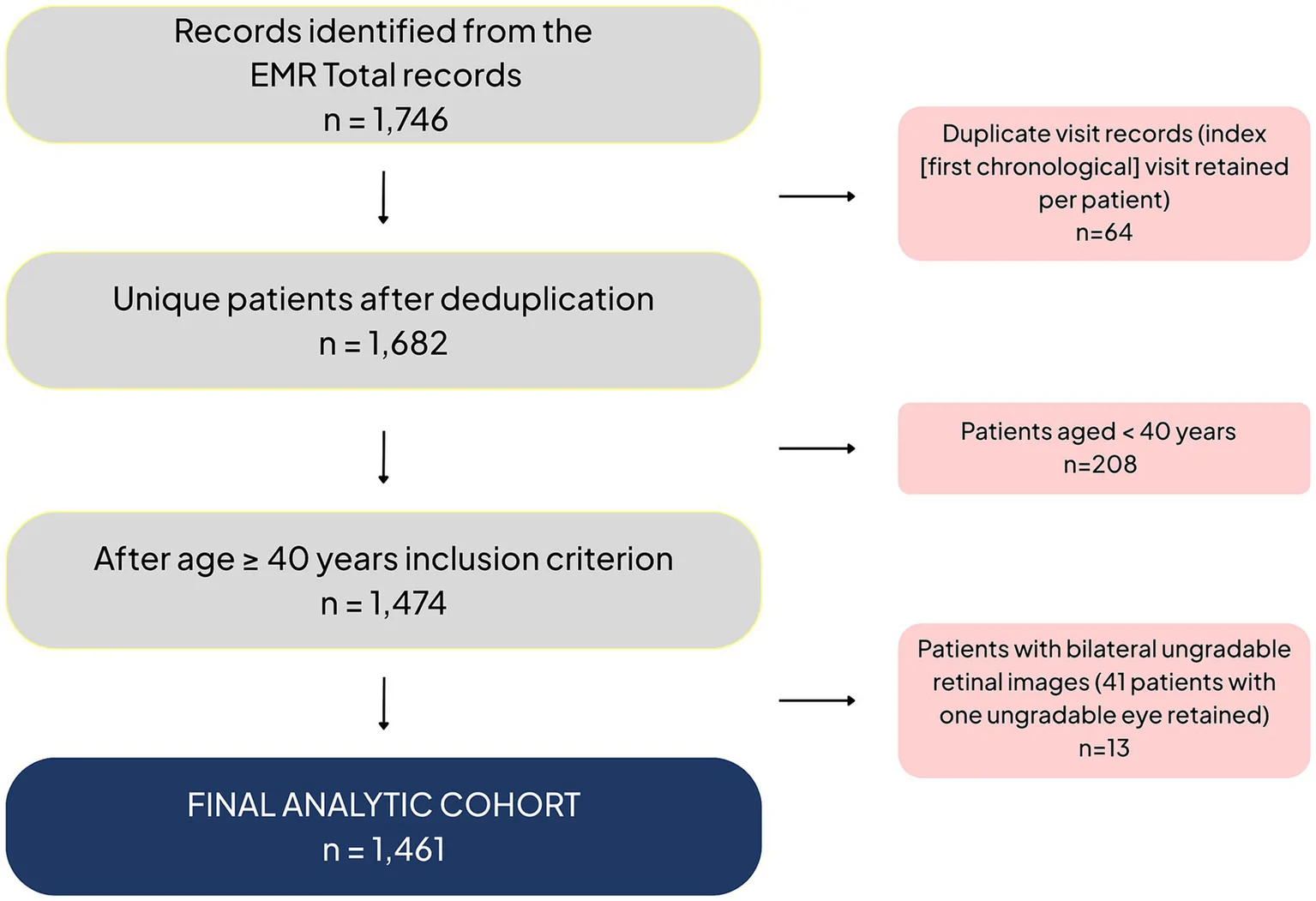
Derivation of the final analytic cohort. Flow diagram shows sequential exclusion of duplicate visit records, patients aged <40 years, and patients with bilateral ungradable images, resulting in the final analytic cohort of 1,461 patients. Distribution across Reti-CVD categories is shown using pre-specified thresholds: low risk (<31), moderate risk (31–41), and high risk (>41).

The primary data source was the electronic medical record (EMR), integrated with the retinal imaging platform output. The raw dataset comprised 1,746 records spanning the study period, which included repeat visits for some patients. Sequential eligibility filtering was applied as follows (Figure 1): duplicate visit records were resolved by retaining the index (first chronological) visit per patient, yielding 1,682 unique patients; 208 patients aged under 40 years were then excluded; and a further 13 patients were excluded owing to bilateral ungradable image quality, resulting in a final analytic cohort of 1,461 patients.

### 2.4 Retinal imaging and AI risk stratification

The Reti-CVD model has been previously validated in the Cardiovascular and Metabolic Diseases Etiology Research Center-High Risk (CMERC-HI) cohort, prospectively, in South Korea (Lee et al., 2024). The validation study included 1,106 participants with available retinal fundus imaging who were followed for 5 years. The primary endpoint was the occurrence of fatal and non-fatal CVD events, including heart failure hospitalization, stroke, myocardial infarction, and cardiovascular mortality. Although the study was validated in the CMERC-HI cohort, the algorithm was not recalibrated for the present Dubai-based cohort, and external validation in populations from Middle Eastern, expatriate, or specialized hybrid clinics has not yet been established.

The manufacturer-defined intended-use population varies by regulatory market but generally comprises adults undergoing cardiovascular risk assessment. For example, in the European Union, where the algorithm is CE marked, the device is indicated for adults over 19 years of age and is intended for use by qualified healthcare professionals in hospitals, clinics, and health-check settings. It may be used for general cardiovascular risk screening, including asymptomatic adults, while being particularly relevant for individuals with cardiometabolic risk factors such as diabetes, hypertension, hyperlipidemia, obesity, or metabolic syndrome. Relevant exclusions include patients in whom an adequate fundus image cannot be obtained, such as those with dense white cataract, as well as non-fundus images, images from unsupported devices, or images that do not meet predefined technical quality requirements. The algorithm includes an integrated image-quality assessment module that evaluates input suitability before cardiovascular risk analysis is performed. The output is intended to support, rather than replace, clinical judgment and should not be used as the sole basis for diagnosis or treatment.

In the present study, retinal fundus images were acquired using an automated, non-mydriatic retinal camera (Topcon 3D OCT-1 Maestro2). Both eyes were imaged at each visit. Images taken were analyzed by the Dr. Noon version 1.0.0 AI platform (Mediwhale Inc., Seoul, South Korea), which is the same version that has been used in the CMERC-HI Korean cohort (Lee et al., 2024). Dr. Noon is a deep learning algorithm that generates a continuous cardiovascular risk score (Reti-CVD score, range 0–100) derived from microvascular retinal features, without requiring patient-reported clinical inputs (Lee et al., 2024). Reti-CVD categories were predefined according to the thresholds reported in the original validation study, where low is less than 0.3093, moderate is between 0.3093 and 0.4076, and high is any value greater than 0.4076 (Lee et al., 2024) corresponding to <31, 31–41, and >41.

Image quality was assessed using the platform’s CNN-based binary quality assessment module, classifying images as gradable or ungradable. Operators also performed manual quality assessment in accordance with manufacturer training recommendations, in which 75% of image details could be assessed by the operator, including vessel branching, microaneurysm lesions, and drusen. Repeat image acquisition followed the routine clinical workflow when image quality was inadequate. When both eyes are gradable, the patient-level Reti-CVD score was calculated as the mean of both eye-specific scores. Patients with one gradable eye were retained because the algorithm can generate a score from a single gradable image, whereas patients with bilateral ungradable images were excluded. Out of 1,461 images, 1,419 (97.1%) had two gradable eyes, while 42 (2.9%) had one gradable eye. In this, 22 (1.5%) and 20 (1.4%) had a gradable left and right eye, respectively.

### 2.5 Clinical and laboratory data

Demographic variables, smoking status, and comorbidity diagnoses were extracted from the EMR. Treatment status for antihypertensive and lipid-lowering agents was recorded. Anthropometric measurements (BMI, weight) and blood pressure were obtained at the index visit. Laboratory data, including HbA1c, lipid panel (LDL, HDL, total cholesterol, triglycerides), renal function (eGFR, creatinine), liver enzymes (ALT, AST), C-reactive protein (CRP), uric acid, albumin, and fasting glucose, were extracted from the most recent measurement at or before the index visit. Structured race/ethnicity or nationality fields were not consistently available in the retrospective extract and therefore could not be used for subgroup calibration or fairness analyses.

### 2.6 PREVENT scores

The PREVENT equation was implemented to compute individualized 10-year CVD risk scores for all eligible patients in the SAFER cohort. It was applied to patients aged 40–79 years, as per the validated age range reported in the original development paper (Khan et al., 2024). Continuous inputs were log-transformed and centered on the development cohort mean covariate pattern as published in the Supplementary material of Khan et al. (2024). Sex-specific baseline survival values at 10 years (S₀ (10)) of 0.9742 for females and 0.9641 for males were applied. These correspond to a baseline 10-year CVD risk of 2.58% in women and 3.59% in men at mean covariate values. The final risk score was expressed as a percentage (10-year absolute CVD risk). Risk categories were defined using two classification schemes: the standard AHA 4-tier scheme (Low <3%, Borderline 3–5%, Intermediate 5–7.5%, High ≥7.5%) and a collapsed 3-tier scheme (Low <5%, Moderate 5–10%, High ≥10%) designed to permit direct agreement comparison with the Reti-CVD Low/Moderate/High categories. PREVENT was treated as a comparator equation and not as a gold standard for clinical events or subclinical vascular disease.

### 2.7 Statistical analysis

All statistical analyses were performed using Stata version 17 (StataCorp LLC, College Station, TX). Given the exploratory nature of this implementation study, analyses were intended to characterize associations and agreement patterns rather than develop or validate a predictive model. Normality of continuous variables was assessed by the Shapiro–Wilk test; all variables deviated significantly from a normal distribution (W statistics ranging from 0.29 to 0.99; all *p* < 0.05) and are therefore reported as median with interquartile range (IQR). Categorical variables are reported as frequency and percentage.

Differences in baseline characteristics across the three Reti-CVD risk categories were evaluated using the Kruskal-Wallis test for continuous variables and the Pearson chi-square test for categorical variables. For continuous variables yielding a statistically significant Kruskal-Wallis result, pairwise post-hoc comparisons (Low vs. Moderate, Low vs. High, and Moderate vs. High) were conducted using the Wilcoxon rank-sum test with Bonferroni correction for multiplicity; adjusted *p*-values are reported (significance threshold: *p* < 0.017, corresponding to an overall *α* of 0.05). Missing data are reported by variable; complete-case analysis was applied throughout, and no multiple imputation was performed for the primary descriptive analysis. A two-sided significance level of *p* < 0.05 was applied for all primary comparisons.

Alignment of the continuous Reti-CVD score against PREVENT-defined binary outcomes was quantified using the area under the receiver operating characteristic curve (AUC), evaluated at each standard PREVENT threshold (3, 5, 7.5, 10%). The AUC represents the probability that a randomly selected PREVENT-positive patient has a higher Reti-CVD score than a randomly selected PREVENT-negative patient. Agreement between the PREVENT 3-tier classification and Reti-CVD 3-tier classification was assessed using Cohen’s unweighted Kappa and quadratic-weighted Kappa. Subgroup agreement was evaluated by sex and age group (<50 vs. ≥ 50 years). All analyses were restricted to the 1,224 patients with complete PREVENT score data (84.0% of the analytic cohort).

### 2.8 Missing data

The completeness of clinical and laboratory data varied by variable type. Age, sex, Reti-CVD score, and risk group classification were available for all 1,461 participants. Comorbidity status flags had negligible missingness (<0.3%). Routine laboratory measurements had moderate missingness, ranging from 3.3% for BMI to 15.2% for CRP, consistent with the expected incompleteness of data derived from real-world clinical records rather than mandated study protocols. Fasting glucose (59.7% missing) and diabetes duration (75.5% missing) had high rates of non-availability; variables with greater than 50% missingness were excluded from primary analyses and are reported only descriptively where available.

PREVENT scores could not be computed for 237 patients (16.2%), primarily because of missing eGFR (181/1,461; 12.4%) and missing cholesterol panel values (148/1,461; 10.1%), both of which are required inputs to the PREVENT equation. To assess possible selection bias, patients with complete versus incomplete PREVENT data were compared across age, sex, Reti-CVD risk distribution, Reti-CVD score, and key cardiometabolic variables. 1,419 (97.1%) of the total cohort had two gradable eyes, while 42 (2.9%) had one gradable eye. Out of those, 22 (1.5%) and 20 (1.4%) had a gradable left and right eye, respectively.

## 3 Results

### 3.1 Cohort characteristics and risk group distribution

Of 1,746 clinical records identified, 1,461 patients met eligibility criteria and were included in the final analytic cohort (Figure 1). The cohort was predominantly young, with a median age of 46 years (IQR 43–52), and comprised a majority of male patients (858, 58.7%). Two-thirds of participants (972, 66.5%) were aged 40–49 years, with a smaller proportion aged 50–59 years (371, 25.4%), 60–69 years (96, 6.6%), and 70 years or older (22, 1.5%). Based on the Reti-CVD algorithm output, 649 patients (44.4%) were categorized as Low risk, 611 (41.8%) as Moderate risk, and 201 (13.8%) as High risk.

### 3.2 Baseline characteristics by Reti-CVD risk category

Baseline demographic, clinical, and biochemical characteristics stratified by Reti-CVD risk category are presented in Table 1. Statistically significant differences were observed across all cardiometabolic domains except CRP and smoking status.

**Table 1:** Baseline characteristics of the analytic cohort stratified by Reti-CVD risk category (*N* = 1,461).

Characteristic	Low (*n* = 649)	Moderate (*n* = 611)	High (*n* = 201)	*p* value
Demographics				
Age, years	43 (41–47)	48 (44–53)	56 (51–63)	<0.001
Male sex, *n* (%)	290 (44.7)	411 (67.3)	157 (78.1)	<0.001
BMI, kg/m^2^	26.1 (23.7–29.5)	27.5 (25.0–30.7)	27.9 (25.2–31.8)	<0.001
Weight, kg	74.3 (64.3–85.4)	80.8 (71.0–93.6)	81.0 (71.5–93.6)	<0.001
Glycaemic markers				
HbA1c, %	5.3 (5.1–5.7)	5.5 (5.2–6.0)	5.9 (5.3–6.8)	<0.001
Diabetes mellitus, *n* (%)	111 (17.2)	162 (26.6)	85 (42.3)	<0.001
Newly diagnosed	43 (6.6)	59 (9.7)	39 (19.4)	
Duration <1 year	23 (3.5)	33 (5.4)	13 (6.5)	
Duration 1–<3 years	35 (5.4)	52 (8.5)	26 (12.9)	
Duration ≥3 years	10 (1.5)	18 (2.9)	7 (3.5)	
Lipid profile				
LDL cholesterol, mg/dL	129.0 (105.1–155.8)	128.0 (97.5–155.1)	117.7 (86.0–148.4)	0.021
HDL cholesterol, mg/dL	54.7 (45.1–66.0)	51.5 (42.0–60.8)	47.6 (41.5–59.4)	<0.001
Total cholesterol, mg/dL	195.6 (169.7–222.5)	195.0 (164.3–219.6)	183.1 (149.2–217.8)	0.011
Triglycerides, mg/dL	90.8 (62.0–133.1)	100.6 (73.5–155.0)	111.2 (81.0–154.7)	<0.001
Lipid-lowering therapy, *n* (%)	246 (38.0)	362 (59.3)	138 (68.7)	<0.001
Blood pressure and renal function				
Systolic BP, mmHg	113 (105–123)	118 (111–127)	125 (114–135)	<0.001
Diastolic BP, mmHg	74 (68–81)	76 (71–83)	78 (72–85)	<0.001
eGFR, mL/min/1.73m^2^	101.7 (90.0–115.4)	103.9 (90.3–120.2)	100.2 (86.4–113.9)	0.046
Creatinine, mg/dL	0.8 (0.7–0.9)	0.9 (0.7–1.0)	0.9 (0.8–1.0)	<0.001
Cardiovascular comorbidities				
Hypertension, *n* (%)	86 (13.3)	139 (22.8)	73 (36.3)	<0.001
Antihypertensive therapy, *n* (%)	54 (8.4)	126 (20.7)	70 (34.8)	<0.001
Hyperlipidemia, *n* (%)	448 (69.2)	488 (80.0)	163 (81.1)	<0.001
Retinopathy, *n* (%)	9 (1.4)	8 (1.3)	9 (4.5)	0.008
Metabolic syndrome, *n* (%)	31 (4.8)	19 (3.1)	3 (1.5)	0.062
CKD, *n* (%)	3 (0.5)	1 (0.2)	3 (1.5)	0.061
Inflammatory and hepatic markers				
CRP, mg/dL	0.1 (0.1–0.3)	0.1 (0.1–0.3)	0.1 (0.1–0.3)	0.776
ALT, U/L	19.1 (13.9–27.8)	23.3 (16.8–33.3)	22.7 (16.9–29.9)	<0.001
AST, U/L	18.9 (15.4–23.9)	20.3 (16.8–25.1)	19.1 (16.8–23.9)	0.003
Uric acid, mg/dL	4.9 (4.0–5.9)	5.4 (4.4–6.4)	5.6 (4.7–6.7)	<0.001
Smoking status				
Never smoked, *n* (%)	479 (76.5)	431 (73.9)	136 (72.0)	0.565
Current smoker, *n* (%)	113 (18.1)	119 (20.4)	44 (23.3)	
Former smoker, *n* (%)	34 (5.4)	33 (5.7)	9 (4.8)	
Reti-CVD algorithm outputs				
Reti-CVD score	26.1 (22.9–28.7)	35.3 (33.0–37.9)	43.7 (42.3–46.3)	<0.001
AI-predicted vascular age, years	39.7 (37.1–43.2)	45.6 (41.0–51.6)	55.7 (51.0–61.5)	<0.001

Data are presented as median (interquartile range) for continuous variables and *n* (%) for categorical variables. *p* values are from the Kruskal-Wallis test (continuous) and Pearson chi-square test (categorical). Lipid-lowering therapy: proportion among all participants in each risk group. Diabetes duration categories: reported among patients with duration data available only (*n* = 358 total); missing *n* = 1,103. Metabolic syndrome defined per the 2009 harmonized IDF/AHA/NHLBI criteria. AI-predicted vascular age: retinal biological age estimate generated by the Dr. Noon algorithm; distinct from chronological age. BMI = body mass index; BP = blood pressure; CKD = chronic kidney disease; CRP = C-reactive protein; eGFR = estimated glomerular filtration rate; HbA1c = glycated hemoglobin; HDL = high-density lipoprotein; IQR = interquartile range; LDL = low-density lipoprotein.

#### 3.2.1 Age and sex

Age demonstrated the most pronounced and consistent gradient across risk categories, with median ages of 43, 48, and 56 years in the Low, Moderate, and High groups, respectively (Kruskal-Wallis *p* < 0.001). Pairwise post-hoc analysis confirmed that each successive risk tier was significantly older than the preceding one (all Bonferroni-adjusted *p* < 0.001; Table 2). Male sex was substantially more prevalent in higher-risk groups, rising from 44.7% in the Low-risk group to 67.3% in the Moderate-risk and 78.1% in the High-risk group (*p* < 0.001).

**Table 2:** Pairwise post-hoc comparisons of selected continuous variables across Reti-CVD risk groups.

Variable	Low vs. Moderate adj. P	Low vs. High adj. P	Moderate vs. High adj. P	Overall P
Age	**<0.001**	**<0.001**	**<0.001**	<0.001
BMI^ᵃ^	**<0.001**	**<0.001**	0.447	<0.001
HbA1c	**<0.001**	**<0.001**	**<0.001**	<0.001
LDL cholesterol^ᵇ^	0.907	**0.014**	0.145	0.021
HDL cholesterol^ᵃ^	**<0.001**	**<0.001**	0.386	<0.001
Systolic BP^ᶜ^	**<0.001**	**<0.001**	**<0.001**	<0.001
Diastolic BP^ᵃ^	**<0.001**	**<0.001**	0.231	<0.001
eGFR^ᵈ^	0.259	0.696	0.075	0.046
Reti-CVD score	**<0.001**	**<0.001**	**<0.001**	<0.001

Values are Bonferroni-adjusted *p* values from Wilcoxon rank-sum post-hoc comparisons. Overall *p* values are from the Kruskal-Wallis test across all three Reti-CVD risk groups. Statistical significance after Bonferroni correction was defined as adjusted *p* < 0.017. BP = blood pressure; eGFR = estimated glomerular filtration rate; HbA1c = glycated hemoglobin; HDL = high-density lipoprotein; LDL = low-density lipoprotein. Bold value means Bonferroni-significant (adj. *p* < 0.017).

ᵃ Moderate and High groups did not differ significantly; Low risk differed from both.

ᵇ Inverse gradient; only the Low vs High comparison was significant, consistent with confounding by lipid-lowering treatment.

ᶜ All three pairwise comparisons were significant; systolic BP uniquely discriminated each successive risk tier.

ᵈ Non-monotonic pattern; overall significance was not driven by a consistent directional gradient, and no pairwise comparison reached significance.

#### 3.2.2 Glycemic status

HbA1c increased in a stepwise fashion across risk categories (5.3, 5.5, and 5.9% in the Low, Moderate, and High groups, respectively; *p* < 0.001), with all pairwise comparisons reaching significance after correction (Table 2). The prevalence of diabetes mellitus rose markedly with increasing Reti-CVD risk, from 17.2% in the Low-risk group to 26.6% in the Moderate-risk and 42.3% in the High-risk group (*p* < 0.001). Among patients with diabetes, newly diagnosed diabetes was numerically more frequent in the High Reti-CVD group; however, diabetes-duration data had substantial missingness and should be interpreted descriptively rather than as a primary inferential finding.

#### 3.2.3 Lipid profile

An inverse relationship between LDL cholesterol and Reti-CVD risk group was observed (Low: 129.0 mg/dL, Moderate: 128.0 mg/dL, High: 117.7 mg/dL; overall *p* = 0.021). Post-hoc analysis revealed that this difference was driven primarily by the Low versus High comparison (adjusted *p* = 0.014), with no significant difference between the Low and Moderate groups (adjusted *p* = 0.907) or the Moderate and High groups (adjusted *p* = 0.145). This inverse pattern is consistent with ‘confounding by treatment’: lipid-lowering therapy was substantially more prevalent in higher-risk patients, prescribed in 38.0, 59.3, and 68.7% of the Low, Moderate, and High groups, respectively (*p* < 0.001). HDL cholesterol declined progressively from the Low to the Moderate and High groups (54.7, 51.5, and 47.6 mg/dL, respectively; *p* < 0.001), with both the Low vs. Moderate and Low vs. High comparisons significant after Bonferroni correction, but no significant difference was observed between the Moderate and High groups (adjusted *p* = 0.386). Triglycerides showed a monotonic increase with the risk group (90.8, 100.6, and 111.2 mg/dL; *p* < 0.001).

#### 3.2.4 Blood pressure

Systolic blood pressure increased progressively across risk categories (113, 118, and 125 mmHg in the Low, Moderate, and High groups; *p* < 0.001) and was the only cardiometabolic variable for which all three pairwise post-hoc comparisons remained statistically significant after Bonferroni correction (Table 2). Diastolic blood pressure followed a similar overall gradient (74, 76, and 78 mmHg; *p* < 0.001), though the Moderate versus High comparison did not reach significance after correction (adjusted *p* = 0.231).

#### 3.2.5 Comorbidities and treatment burden

Hypertension prevalence increased significantly with Reti-CVD risk category (13.3, 22.8, and 36.3% in the Low, Moderate, and High groups; *p* < 0.001), as did antihypertensive treatment use (8.4, 20.7, and 34.8%; *p* < 0.001). Hyperlipidemia was common across all groups (69.2, 80.0, and 81.1%; *p* < 0.001), with a significant difference between Low and the two higher-risk groups, but no significant difference between Moderate and High (*p* < 0.001 for the overall comparison). Retinopathy was significantly more prevalent in the High-risk group (4.5%) compared with both the Low (1.4%) and Moderate (1.3%) groups (*p* = 0.008). Metabolic syndrome demonstrated a counterintuitive inverse gradient (4.8, 3.1, and 1.5% across Low, Moderate, and High; *p* = 0.062). CKD and smoking status did not differ significantly across risk categories (*p* = 0.061 and *p* = 0.565, respectively).

#### 3.2.6 Inflammatory markers

C-reactive protein did not differ significantly across the three Reti-CVD risk groups (median 0.1 mg/dL in all groups; *p* = 0.776). Uric acid showed a significantly increasing gradient (4.9, 5.4, and 5.6 mg/dL; *p* < 0.001). ALT and AST also differed significantly across groups (*p* < 0.001 and *p* = 0.003, respectively).

#### 3.2.7 AI-predicted vascular age

The AI-predicted vascular age, a secondary output of the Reti-CVD algorithm reflecting the estimated ‘biological age’ of the retinal microvasculature, increased markedly across risk categories: 39.7 years (IQR 37.1–43.2) in the Low-risk group, 45.6 years (41.0–51.6) in the Moderate-risk group, and 55.7 years (51.0–61.5) in the High-risk group (*p* < 0.001 for all pairwise comparisons). Notably, in the High-risk group, the median AI-predicted vascular age (55.7 years) closely approximated the median chronological age of 56 years.

**Figure 2:**
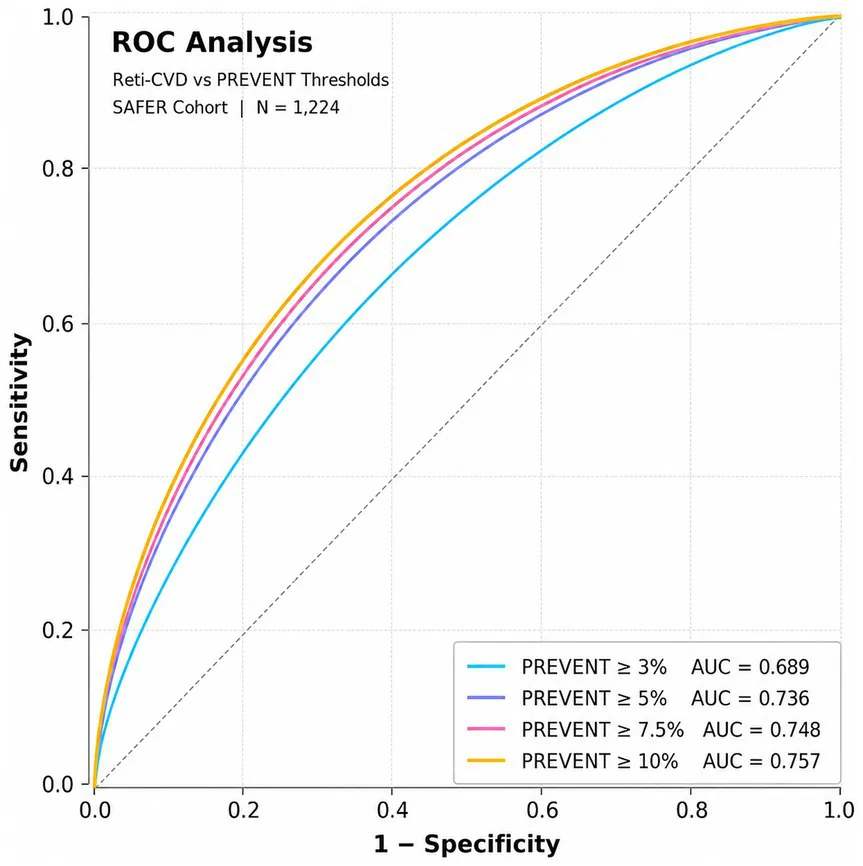
Receiver operating characteristic (ROC) curves for Reti-CVD discrimination of PREVENT-defined risk thresholds. AUC values quantify discrimination against PREVENT equation thresholds (> = 3%, > = 5%, > = 7.5%, and > = 10%) and should not be interpreted as diagnostic or prognostic performance against observed ASCVD events.

To characterise the divergence between vascular and chronological ageing at the individual level, a per-patient delta (AI vascular age minus chronological age) was computed for each participant (Figure 2). This analysis revealed a progressive and statistically significant narrowing of the vascular-chronological age gap across risk tiers. Low-risk patients had a median vascular age 4.1 years younger than their chronological age (IQR − 6.1 to −1.8 years), suggesting well-preserved retinal microvascular structure relative to age. In Moderate-risk patients, this gap narrowed to −2.6 years (IQR − 5.2 to +0.5 years), reflecting early microvascular aging beginning to close the deficit. In High-risk patients, the delta reached −1.5 years (IQR − 4.4 to +1.6 years), the only group whose interquartile range crosses zero, meaning that vascular age routinely meets or exceeds chronological age in this subgroup. All three pairwise comparisons were significant after Bonferroni correction (Low vs. Moderate and Low vs. High: both adjusted *p* < 0.001; Moderate vs. High: adjusted *p* = 0.004).

### 3.3 Factors associated with high Reti-CVD risk categorization

#### 3.3.1 Univariable analysis

In univariable analysis (Table 3), age showed the strongest association with High Reti-CVD risk (OR 3.79 per SD, 95% CI 3.18–4.52; *p* < 0.001), followed by systolic blood pressure (OR 1.86; *p* < 0.001). Female sex was strongly protective (OR 0.35, 95% CI 0.25–0.50; *p* < 0.001). Hypertension (OR 2.62) and diabetes (OR 2.64) each conferred approximately 2.6-fold greater unadjusted odds (both *p* < 0.001). LDL showed an inverse association (OR 0.81; *p* = 0.009), consistent with confounding by lipid-lowering treatment.

**Table 3:** Logistic regression: independent predictors of high Reti-CVD risk (overall model subgroup stratification).

Variable	Panel A — overall multivariable model (*N* = 1,122; events = 156)					Panel B — sex- and age-stratified adjusted ORs		
	Unadj. OR	95% CI	Adj. OR†	95% CI	P (adj.)	Male OR (*N* = 704; ev = 125)	Female OR (*N* = 469; ev = 35)	Age≥50 OR (*N* = 391; ev = 134)
Demographic								
Age (per 1 SD, ≈7 yr)	3.79	3.18–4.52	**3.99**	**3.17–5.04**	**<0.001**	**5.16 (3.81–6.99)**	**2.80 (1.89–4.14)**	—
Female sex (ref: Male)	0.35	0.25–0.50	**0.20**	**0.11–0.36**	**<0.001**	—	—	**0.29 (0.16–0.52)**
BMI (per 1 SD)	1.26	1.09–1.45	1.02	0.80–1.30	0.887	1.13 (0.85–1.50)	1.06 (0.67–1.68)	0.96 (0.74–1.24)
Smoking (ref: never)								
Current smoker	1.28	0.89–1.84	**1.83**	**1.10–3.06**	**0.021**	**2.35 (1.34–4.10)**	0.42 (0.07–2.51)	**2.31 (1.29–4.15)**
Former smoker	0.85	0.42–1.74	0.96	0.35–2.63	0.942	—	—	—
Cardiometabolic								
Hypertension	2.62	1.90–3.61	1.44	0.88–2.36	0.145	—	—	—
Diabetes mellitus	2.64	1.94–3.60	1.09	0.61–1.95	0.780	—	—	—
HbA1c (per 1 SD)	1.50	1.32–1.71	1.20	0.96–1.50	0.118	1.10 (0.84–1.43)	**1.82 (1.12–2.93)†**	1.08 (0.84–1.39)
Lipid Profile								
LDL cholesterol (per 1 SD)	0.81	0.69–0.95	1.03	0.83–1.28	0.795	—	—	—
HDL cholesterol (per 1 SD)	0.78	0.66–0.93	0.97	0.73–1.28	0.826	—	—	—
Triglycerides (per 1 SD)	1.18	1.02–1.35	0.94	0.74–1.20	0.626	—	—	—
Hemodynamic & renal								
Systolic BP (per 1 SD)	1.86	1.59–2.16	1.13	0.89–1.44	0.304	1.02 (0.76–1.39)	1.50 (1.00–2.27)	1.48 (1.16–1.88)*
eGFR (per 1 SD)	0.84	0.71–0.98	0.83	0.67–1.04	0.107	0.80 (0.62–1.03)	0.65 (0.38–1.09)	0.76 (0.59–0.97)*
CRP (per 1 SD)	1.12	0.99–1.27	1.14	0.98–1.33	0.084	—	—	—

Panel A model fit: *N* = 1,122 | Events = 156 | McFadden R^2^ = 0.345 | Hosmer-Lemeshow *p* = 0.856 | Mean VIF = 1.38 | Panel B: no interaction *p* < 0.05; age < 50 model not shown (LR P = 0.105, 26 events). All continuous predictors standardized to mean 0, SD 1 before entry; ORs represent change per 1-SD increase. † Adjusted model (Panel A): all variables entered simultaneously, complete-case analysis. Blue shading = *p* < 0.05 in adjusted model. * *p* < 0.05 within the stratum-specific model (Panel B). † *p* < 0.05 in the female-specific subgroup model (HbA1c). — = not applicable in that stratum (predictor collinear with group definition, or model not estimated). Int. P = interaction *p*-value (Wald test, Stata testparm); all interaction *p* > 0.09. Panel B restricted to key predictors entered in stratified models. Hypertension, Diabetes, Lipids, and CRP not shown in Panel B (non-significant across all strata; Panel B models not powered for these effects). Blue shading means statistical significance (*p* < 0.05) in the adjusted model (Age, Female sex, Current smoking).

#### 3.3.2 Adjusted multivariable model

The primary adjusted model was fitted on 1,122 complete cases (156 High-risk events, 13.9%). The model showed excellent calibration (Hosmer-Lemeshow *p* = 0.856), good overall fit by McFadden pseudo-R^2^ of 0.345, and no multicollinearity (mean VIF = 1.38). After mutual adjustment, three independent predictors remained: age (OR 3.99 per SD, 95% CI 3.17–5.04; *p* < 0.001), female sex (OR 0.20, 95% CI 0.11–0.36; *p* < 0.001), and current smoking (OR 1.83, 95% CI 1.10–3.06; *p* = 0.021). Hypertension (*p* = 0.145), diabetes (*p* = 0.780), and HbA1c (*p* = 0.118) became non-significant after adjustment, reflecting confounding by age and sex. The inverse LDL association disappeared (adjusted OR 1.03), confirming treatment confounding.

#### 3.3.3 Sensitivity analyses

The restricted model (*N* = 1,196; excluding CRP and eGFR) yielded identical conclusions: age (OR 4.27), female sex (OR 0.21), and current smoking (OR 1.83) remained the only significant predictors. The diabetic-patient model (*N* = 322) confirmed age and female sex as dominant predictors; diabetes duration was non-significant (*p* = 0.988).

### 3.4 Subgroup and sensitivity analysis

#### 3.4.1 Sex and age stratification

##### 3.4.1.1 Sex-stratified models

In men (N = 704; 125 events), age (OR 5.16 per SD, 95% CI 3.81–6.99; *p* < 0.001) and current smoking (OR 2.35, 95% CI 1.34–4.10; *p* = 0.003) were independent predictors. In women (N = 469; 35 events), age (OR 2.80; *p* < 0.001) and HbA1c (OR 1.82 per SD, 95% CI 1.12–2.93; *p* = 0.015) were significant. The sex×HbA1c interaction was non-significant (*p* = 0.452), reflecting limited power (35 High-risk female events) rather than confirmed effect modification.

##### 3.4.1.2 Age-stratified models

In patients aged under 50 (*N* = 782; 26 events), the overall model was non-significant (LR *p* = 0.105). In patients aged ≥50 (*N* = 391; 134 events), four predictors emerged: female sex (OR 0.29; *p* < 0.001), current smoking (OR 2.31; *p* = 0.005), systolic BP (OR 1.48; *p* = 0.001), and eGFR (OR 0.76; *p* = 0.025), consistent with progressive haemodynamic and renal contribution beyond age 50. No interaction term was significant (all *p* > 0.09).

#### 3.4.2 Preexisting conditions and Reti-CVD risk categorization

##### 3.4.2.1 Traditional marker profile

Among 212 patients meeting both LDL < 100 mg/dL and BP < 130/80 mmHg, 120 (56.6%) were Moderate or High Reti-CVD risk. Reti-CVD distribution did not differ significantly between traditionally controlled and non-controlled patients (*p* = 0.843), suggesting that conventional biomarker control was not consistently aligned with retinal AI-derived risk in this cohort.

##### 3.4.2.2 Patients without preexisting conditions

Among 326 patients without any preexisting HTN or HLD, 143 (43.9%) were Reti-CVD Moderate or High. These patients had higher HbA1c (5.4 vs. 5.2%; *p* = 0.0001), systolic BP (115 vs. 108 mmHg; *p* < 0.001), and BMI (27.1 vs. 25.1 kg/m^2^; *p* = 0.002) compared with Low-risk patients, while LDL, eGFR, and CRP did not differ, suggesting a retinal microvascular risk phenotype driven by glycemic and hemodynamic burden.

##### 3.4.2.3 Preexisting-condition group

Among 1,132 patients with preexisting HTN or HLD, the 166 High Reti-CVD patients had higher HbA1c (5.9 vs. 5.5%; *p* < 0.001), systolic BP (125 vs. 117 mmHg; *p* < 0.001), and BMI (*p* = 0.008). LDL was lower (122 vs. 136 mg/dL; *p* = 0.003), reflecting more intensive lipid-lowering therapy (Table 4).

**Table 4:** Cardiometabolic parameters by Reti-CVD risk within preexisting-condition subgroups.

Parameter	HTN/HLD high (*n* = 166)	HTN/HLD Low+Mod (*n* = 966)	No HTN/HLD Mod+High vs. Low	*p*
With preexisting HTN/HLD (*n* = 1,132)				
HbA1c, %	5.9 (5.3–6.8)	5.5 (5.2–5.9)	5.4 vs. 5.2	<0.001/0.0001
LDL, mg/dL‡	122 (88–151)	136 (106–160)	111 vs. 112	0.003/0.957
Systolic BP, mmHg	125 (114–134)	117 (109–126)	115 vs. 108	<0.001 / <0.001
BMI, kg/m^2^	28.2	27.1	27.1 vs. 25.1	0.008/0.002
eGFR, mL/min/1.73m^2^	101 (89–114)	103 (90–118)	99 vs. 102	0.130/0.439
CRP, mg/dL	0.13	0.14	0.13 vs. 0.12	0.832/0.916

Data are median (IQR). *p* values: Wilcoxon rank-sum. P column: A = HTN/HLD group / B=No-HTN/No-HLD group. ^‡^Lower LDL in High Reti-CVD reflects more intensive lipid-lowering therapy.

### 3.5 Comparison with the PREVENT equation

#### 3.5.1 PREVENT score distribution

**Figure 3:**
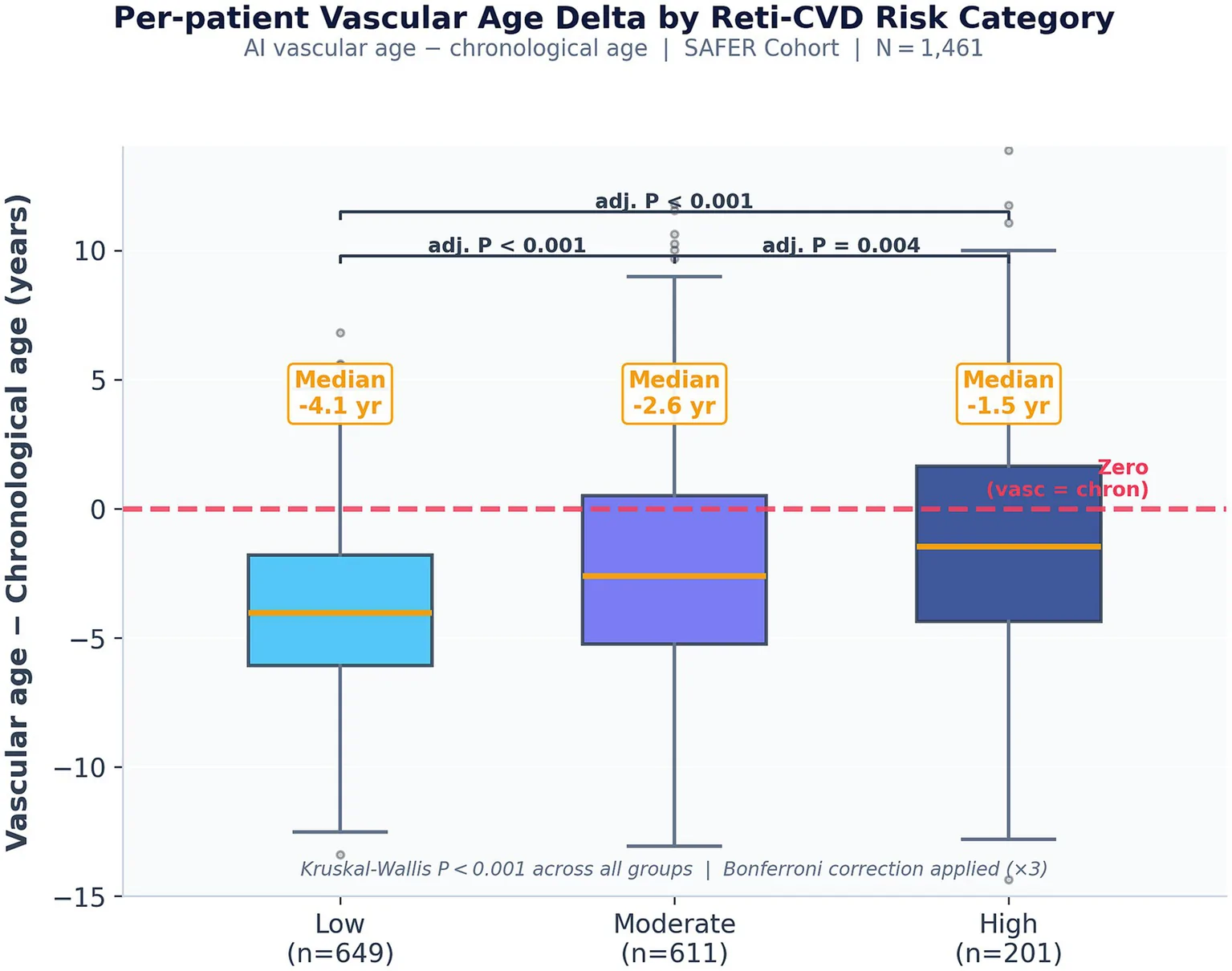
Per-patient vascular age delta by Reti-CVD risk category (*N* = 1,461). Vascular age delta was calculated as AI-predicted vascular age minus chronological age. Boxes represent interquartile range (IQR); orange line indicates median; whiskers extend to 1.5 × IQR. The dashed red line represents zero difference between vascular and chronological age.

Valid PREVENT 10-year CVD risk scores were computed for 1,224 of 1,461 patients (83.8%; 237 excluded due to missing eGFR [12.4%] or cholesterol [10.1%]). The cohort had low overall PREVENT risk (median 2.37%, IQR 1.65–3.72%), consistent with its young median age. PREVENT scores increased monotonically across Reti-CVD risk groups (Low 1.93%, Moderate 2.54%, High 4.15%; Kruskal-Wallis *p* < 0.001) (Figure 3). Using standard AHA 4-tier classification, 64.1% were PREVENT Low (<3%), 17.9% Borderline, 7.6% Intermediate, and 10.5% High (≥7.5%) (see Table 5).

**Table 5:** Clinical characteristics by PREVENT 4-tier category (*N* = 1,224).

Variable	Low (<3%) (*n* = 784)	Borderline (3–5%) (*n* = 219)	Intermediate (5–7.5%) (*n* = 93)	High (≥7.5%) (*n* = 128)	*p*
Age, years	45 (42–50)	46 (43–53)	49 (45–55)	52 (45–57)	<0.001
HbA1c, %	5.3 (5.1–5.6)	5.6 (5.3–6.3)	5.7 (5.3–6.8)	6.6 (5.9–7.6)	<0.001
Systolic BP, mmHg	113 (106–122)	122 (113–130)	122 (115–134)	127 (117–142)	<0.001
BMI, kg/m^2^	26.2	28.2	28.3	27.8	<0.001
eGFR, mL/min/1.73m^2^	101 (89–115)	106 (93–123)	102 (89–124)	104 (89–120)	0.007
**Reti-CVD score**	30.2 (25–35)	32.8 (28–39)	36.7 (32–40)	37.8 (34–43)	**<0.001**
PREVENT 10-yr risk, %	1.9 (1.4–2.3)	3.6 (3.3–4.0)	6.3 (5.8–6.9)	10.3 (8.6–13.2)	<0.001

Data are median (IQR). †Reti-CVD score row shows independent gradient of AI-derived vascular risk across PREVENT tiers (*p* < 0.001). BMI presented as median only without IQR. Blue shading on the Reti-CVD score row = emphasis as the primary comparator outcome, not significance.

#### 3.5.2 Complete-case comparison for PREVENT analyses

Patients with complete PREVENT data were broadly similar to those with incomplete PREVENT data for demographic variables, Reti-CVD category distribution, continuous Reti-CVD score, and measured cardiometabolic markers (Table 6). Complete cases were more likely to have diabetes and hyperlipidemia, likely reflecting more frequent laboratory monitoring among patients with known metabolic disease. Consequently, PREVENT-comparison results should be interpreted within a complete-case framework, while recognizing that the available complete-case subset was not depleted of higher-risk cardiometabolic patients.

**Table 6:** Characteristics of patients with complete versus incomplete PREVENT data.

Variable	PREVENT complete (*n* = 1,224) [*n* with data shown]	PREVENT missing (*n* = 237) [*n* with data shown]	*p* value
Demographic			
Age, years	46.0 (43.0–52.0) [*n* = 1,224]	47.0 (43.0–52.0) [*n* = 237]	0.325
Male sex, *n* (%)	724 (59.2%) [*n* = 1,224]	134 (56.5%) [*n* = 237]	0.455
Clinical variables			
BMI, kg/m^2^	26.9 (24.4–30.1) [*n* = 1,224]	27.5 (24.9–30.8) [*n* = 189/237, 79.7%]	0.185
HbA1c, %	5.4 (5.1–6.0) [*n* = 1,171/1,224]	5.4 (5.2–5.8) [*n* = 78/237, 32.9%]	0.805
Systolic BP, mmHg	117.0 (108.0–127.0) [*n* = 1,224]	117.0 (108.0–125.0) [*n* = 166/237, 70.0%]	0.451
PREVENT equation components — denominator note available; *n* reflects only those missing the other component†			
eGFR, mL/min/1.73m^2^	102.1 (89.7–117.0) [*n* = 1,224]	99.0 (85.0–122.6) [*n* = 56/237, 23.6%]†	0.472
Total cholesterol, mg/dL	194.2 (166.5–221.0) [*n* = 1,224]	187.2 (161.1–216.9) [*n* = 89/237, 37.6%]††	0.325
Reti-CVD			
Reti-CVD score	31.8 (26.7–37.5) [*n* = 1,224]	33.0 (27.4–38.1) [*n* = 237]	0.327
Reti-CVD risk category, *n* (%)			0.501
Low	549 (44.9%)	100 (42.2%)	
Moderate	512 (41.8%)	99 (41.8%)	
High	163 (13.3%)	38 (16.0%)	
Comorbidities — differences reflect monitoring intensity, not risk-based selection‡			
Diabetes, *n* (%)	319 (26.1%) [*n* = 1,224]	39 (16.7%) [*n* = 234/237]	0.002‡
Hyperlipidemia, *n* (%)	947 (77.4%) [*n* = 1,224]	152 (65.0%) [*n* = 234/237]	<0.001‡
Smoking status	No significant difference [*n* = 1,224]	No significant difference [*n* = 174/237, 73.4%]	0.753

Data are median (IQR) unless stated. *p* values: Wilcoxon rank-sum (continuous), Pearson chi-square (categorical), two-sided. ^†^eGFR available in 56/237 (23.6%) of PREVENT-missing patients — those missing cholesterol but not eGFR. Comparison restricted to this subset; interpret with caution given sparse denominator. ^††^Total cholesterol available in 89/237 (37.6%) of PREVENT-missing patients — those missing eGFR but not cholesterol. Comparison likewise restricted. ^‡^ Significant differences in diabetes (*p* = 0.002) and hyperlipidemia (*p* < 0.001) reflect systematic differences in monitoring intensity among patients with established metabolic disease, not cardiovascular risk-based selection. This enrichment of the complete-case subset for patients with higher metabolic burden produces a conservative bias: the 49.1% discordance rate is an underestimate of the true discordance in an unselected population.

#### 3.5.3 Discrimination: AUC analysis

**Figure 4:**
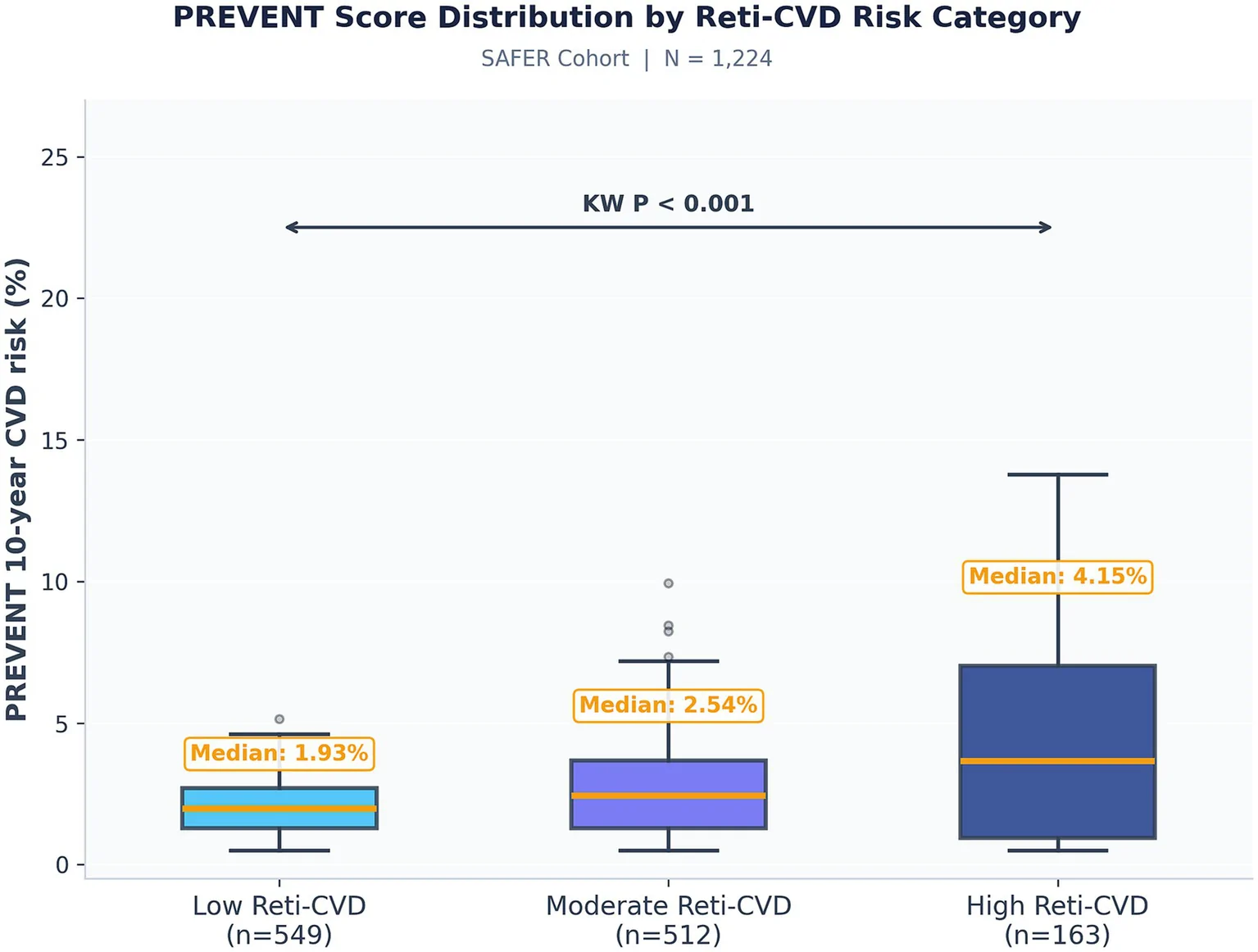
PREVENT score distribution by Reti-CVD risk category. Box plots show PREVENT score distribution across Reti-CVD categories. Boxes represent interquartile range (IQR); orange line indicates median; whiskers extend to 1.5 × IQR.

The Reti-CVD score demonstrated statistically significant discrimination at all four PREVENT thresholds (Figure 4), with AUC increasing from 0.689 (95% CI 0.658–0.719) at ≥3% to 0.757 (95% CI 0.700–0.814) at ≥10%. At the primary statin-eligibility threshold of ≥7.5%, AUC was 0.748 (95% CI 0.705–0.791).

#### 3.5.4 Agreement analysis: Cohen’s kappa

**Figure 5:**
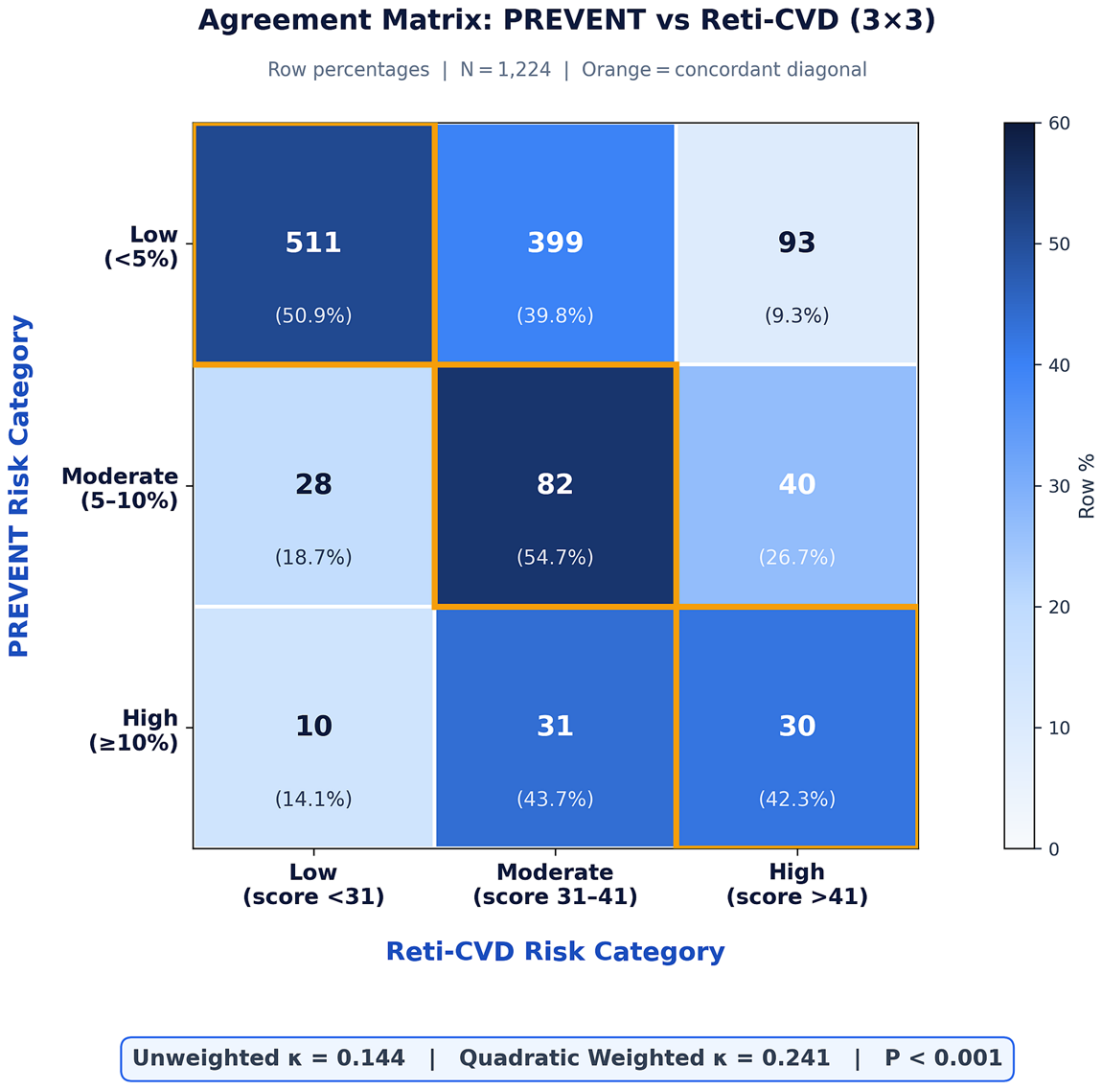
Cross-tabulation of PREVENT and Reti-CVD risk categories (3 × 3, *N* = 1,224). Heatmap shows proportional cross-tabulation between risk classifications. Concordant cells are shown on the diagonal, while off-diagonal cells indicate categorical discordance. This figure describes agreement patterns and does not establish outcome-based categorization benefits.

Overall agreement was 50.9% observed vs. 42.7% expected. Cohen’s unweighted Kappa was 0.144 (slight) and quadratic-weighted Kappa was 0.241 (fair), both *p* < 0.001 (Figure 5; Table 7).

**Table 7:** Subgroup agreement: PREVENT 3-tier vs. Reti-CVD 3-tier.

Subgroup	Unweighted κ	Quadratic weighted κ	Interpretation
**All patients (*N* = 1,224)**	0.144	0.241	Slight/Fair
Male	0.119	0.215	Slight/Fair
Female	0.119	0.173	Slight
Age 40–49 years	0.137	0.162	Slight
Age ≥50 years	0.067	0.176	Slight

κ: <0.20 = Slight | 0.21–0.40 = Fair. All *p* < 0.001.

#### 3.5.5 Discordant risk categorization between PREVENT and Reti-CVD

**Figure 6:**
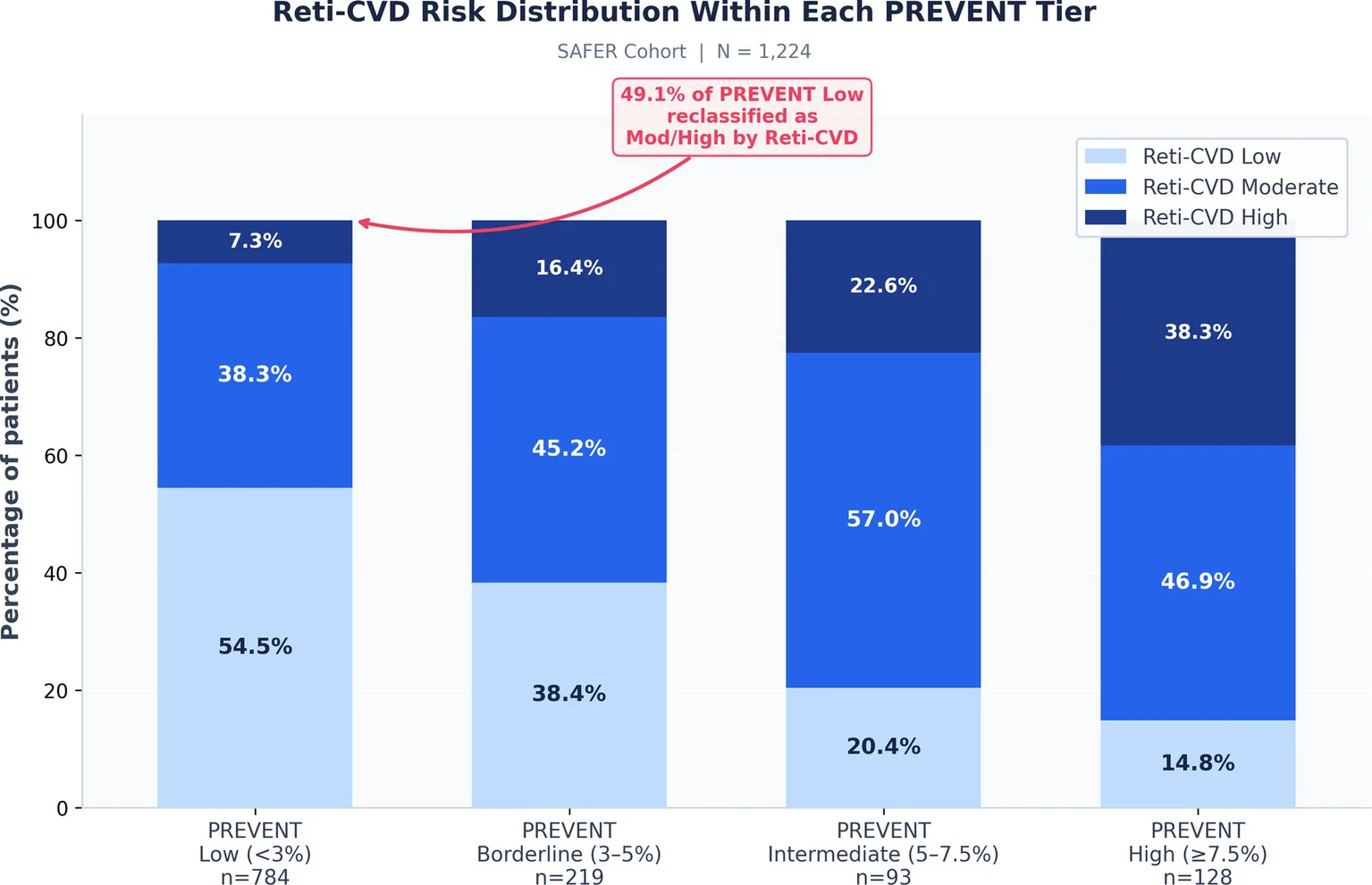
Reti-CVD risk distribution within PREVENT risk tiers (*N* = 1,224). Distribution of Reti-CVD risk categories (low, moderate, high) stratified by PREVENT risk tiers. A proportion of individuals classified as PREVENT low risk were categorized as moderate or high Reti-CVD risk (49.1%). Bars show the proportion of Reti-CVD low, moderate, and high categories within each PREVENT tier.

Among 1,003 PREVENT-Low (<5%) patients, Reti-CVD categorized 399 (39.8%) as Moderate and 93 (9.3%) as High, corresponding to 49.1% categorical discordance towards a higher Reti-CVD category (Figure 6). Among PREVENT <3% (n = 784), 357 (45.5%) were Reti-CVD Moderate or High. Conversely, among 128 PREVENT High (≥7.5%) patients, only 49 (38.3%) were also Reti-CVD High, while 19 (14.8%) were Reti-CVD Low.

#### 3.5.6 Clinical profile across PREVENT tiers

Key cardiometabolic variables showed significant monotonic gradients across PREVENT 4-tier categories (Table 5; all *p* ≤ 0.007). HbA1c rose from 5.3% (PREVENT Low) to 6.6% (PREVENT High). The Reti-CVD score increased monotonically (30.2 → 32.8 → 36.7 → 37.8; *p* < 0.001), confirming broad directional alignment despite low categorical agreement.

## 4 Discussion

This study examined the real-world implementation of the Reti-CVD scan for cardiovascular risk assessment within a hybrid care model. Reti-CVD categories showed clinically plausible associations with cardiometabolic markers, and comparison with the AHA PREVENT equation demonstrated substantial categorical discordance. The stratification of patients into low, moderate, and high Reti-CVD risk categories revealed distinct demographic and clinical differences. Age, diabetes, hypertension, elevated triglycerides, and higher HbA1c levels were all significantly associated with increased Reti-CVD risk. These associations support the biological plausibility of retinal microvascular imaging as a marker associated with cardiometabolic burden, while not establishing prospective event prediction in the present dataset.

The stratification of patients into low, moderate, and high Reti-CVD risk categories revealed distinct demographic and clinical differences. Age, presence of Diabetes, hypertension, elevated triglycerides, and higher HbA1c levels were all significantly associated with increased Reti-CVD risk. These associations support the biological plausibility of the algorithm, given that these factors are well-documented contributors to disease progression (Arnett et al., 2019).

Age and HbA1c showed the strongest gradient across risk categories. At the same time, systolic blood pressure significantly distinguished among the three risk tiers, underscoring the close relationship between retinal vascular structure and systemic vascular injury. However, Male sex was strongly associated with higher Reti-CVD risk, with male prevalence rising from 44.7% in the Low-risk group to 78.1% in the High-risk group. Correspondingly, logistic regression confirmed female sex as an independent protective factor (adjusted OR 0.20; *p* < 0.001), consistent with the well-established male predominance in premature ASCVD and the later cardiovascular risk onset in women relative to men. While C-reactive protein did not differ significantly across Reti-CVD risk groups, this finding is of particular mechanistic interest: it indicates that the Reti-CVD algorithm captures structural microvascular changes associated with long-term atherosclerotic risk, rather than acute or subacute inflammatory processes that elevate circulating CRP.

An inverse relationship was observed between LDL-cholesterol levels and Reti-CVD risk, which requires attention. High-risk patients exhibited lower LDL values, despite having a greater overall cardiometabolic burden (Zhang et al., 2026). This pattern could be partly explained by treatment effects, as lipid-lowering therapy use increased substantially across risk categories. The findings illustrate the challenge of interpreting individual biomarkers in a population receiving preventive therapies. Therefore, including retinal imaging may offer additional insights by capturing structural indications of cumulative vascular damage that are not fully represented by traditional laboratory biomarkers.

The per-patient delta analysis revealed a statistically significant and progressive narrowing of the gap between AI-predicted vascular age and chronological age across Reti-CVD risk tiers. Patients categorized as high risk showed a median vascular age that was nearly the same as their chronological age, while low-risk individuals exhibited significantly younger vascular profiles. This observation suggests that retinal microvascular imaging may reflect aspects of biological vascular ageing. However, the clinical meaning of vascular-age delta, including whether it predicts events or treatment response, remains unproven and requires longitudinal validation.

The comparison between Reti-CVD and PREVENT score provides an insight into the relationship between image-driven retinal score and conventional equation-based risk estimation. Agreement between PREVENT and Reti-CVD score was modest, with an unweighted Kappa of 0.144 and a weighted Kappa of 0.241, although PREVENT scores increased across Reti-CVD risk categories. The level of agreement between the two tools indicates that they assess different aspects of CVD risk. While PREVENT estimates future CVD risk based on established clinical and biochemical risk factors, Reti-CVD directly evaluates current structural microvascular characteristics from retinal images. This difference may also reflect different calibration targets, population mismatch, and category-threshold mismatch. The discordance analysis should be interpreted cautiously. Nearly half of the patients who were classified as low risk by PREVENT (< 5%) were categorized as moderate or high risk by Reti-CVD. Without outcome data, it is not possible to determine whether these patients truly have higher future ASCVD risk, whether Reti-CVD is detecting subclinical vascular injury not represented in PREVENT, or whether the retinal AI thresholds overestimate risk in this population. Conversely, some PREVENT-high patients were categorized as Reti-CVD low, which may reflect lower retinal microvascular abnormality, but may also represent under-classification by the AI tool. These alternatives require validation against clinical endpoints or independent vascular markers. Therefore, these findings cannot establish added information of a distinct biological risk construct.

The findings are particularly relevant for younger cardiometabolic populations because 10-year equation-based CVD risk estimates are often low even when lifetime risk may be substantial (DeFilippis et al., 2017; Selvam et al., 2025). The SAFER cohort had a median age of 46 years and generally low PREVENT risk scores. The observed discordance between Reti-CVD and PREVENT scores is therefore hypothesis-generating for future studies in younger prevention-focused populations, but should not be used alone to infer under- or overtreatment, or outcome benefit in the absence of prospective validation.

The integration of Reti-CVD within a high-engagement metabolic health-focused practice model was both operationally feasible and seamless. Retinal imaging was conducted during standard outpatient visits, and results could be incorporated into clinical review workflows, such as referrals to cardiology and preventive counselling. Whether such deployment improves decision-making, adherence, risk-factor control, or ASCVD outcomes remains to be tested prospectively.

### 4.1 Strengths and limitations

A key strength of this study is its integration of real-world data from a hybrid clinic setting, allowing for the evaluation of the Reti-CVD application within scalable models of care. The cohort size, broad clinical and laboratory characterization, and comparison with PREVENT provide useful preliminary information about feasibility, cardiometabolic associations, and areas of categorical discordance. The analysis also included post-hoc complete-versus-incomplete PREVENT comparisons to assess potential missing-data bias. However, a few limitations are to be considered. Due to the observational, cross-sectional nature of the study, it precludes causal inference and limits the ability to assess predictive accuracy over time. The observational, cross-sectional design precludes causal inference and does not allow assessment of incident ASCVD events, predictive accuracy over time, clinical utility, or outcome-based reclassification benefit. The ROC analyses were performed against PREVENT-defined thresholds rather than clinical endpoints; therefore, AUC values should not be interpreted as diagnostic or prognostic performance. Similarly, low kappa indicates categorical discordance but does not prove that Reti-CVD provides clinically useful added value.

Also, the per-patient vascular age delta represents a single time-point estimate and cannot characterize longitudinal trajectories of retinal microvascular ageing or determine whether the gap narrows or widens with disease progression or treatment. PREVENT scores could not be computed for 16.2% of the cohort owing to missing eGFR or cholesterol data. Although complete-versus-incomplete comparisons did not show significant differences in age, sex, Reti-CVD category, Reti-CVD score, BMI, HbA1c, systolic BP, or eGFR, residual selection bias remains possible. The Dr. Noon/Reti-CVD platform was previously validated in a Korean cohort, while PREVENT was derived from US-based cohorts; neither has been externally validated specifically in this Dubai-based, predominantly expatriate metabolic-clinic population. Structured race/ethnicity data were not consistently available, limiting subgroup calibration and fairness analyses.

## 5 Conclusion

This study demonstrates that AI-based retinal imaging with the Reti-CVD platform was feasible in routine hybrid cardiometabolic care and was associated with clinically plausible gradients in age, diabetes, hypertension, lipid profile, blood pressure, and AI-predicted vascular age. Agreement with PREVENT was slight to fair, indicating substantial categorical discordance between retinal image-derived risk classification and conventional equation-based risk estimation. Many PREVENT-low patients were categorized as moderate or high by Reti-CVD, while some PREVENT-high patients were categorized as low by Reti-CVD; this bidirectional discordance may reflect differences in calibration, threshold selection, population characteristics, or biological domains represented by each approach. These preliminary findings support further prospective validation of retinal AI as a potential complementary assessment tool, but they do not establish improved ASCVD prediction, clinical utility, or outcome-based reclassification benefit.

## Data Availability

The raw data supporting the conclusions of this article will be made available by the authors, without undue reservation.
