# Supplementary material for: AI-powered retinal imaging for early ASCVD risk stratification in a hybrid care model: preliminary findings from SAFER
Source: Front Artif Intell. 2026 Sep 3;9:1920911. doi: 10.3389/frai.2026.1920911 (PMC13582468; doi:10.3389/frai.2026.1920911)
Supplement: Supplementary file 1 [file Table_1.docx]

Supplementary Material

# 1 Supplementary Tables

**Supplementary Table 1: AUC of Reti-CVD Score at Each PREVENT Threshold (N = 1,224)**

| **PREVENT Cutoff** | **Cases** | **Non-cases** | **Total N** | **AUC** | **95% CI** |
| --- | --- | --- | --- | --- | --- |
| ≥3% | 440 | 784 | 1,224 | 0.689 | 0.658–0.719 |
| ≥5% | 221 | 1,003 | 1,224 | 0.736 | 0.701–0.771 |
| ≥7.5% | 128 | 1,096 | 1,224 | 0.748 | 0.705–0.791 |
| **≥10%** | 71 | 1,153 | 1,224 | **0.757** | 0.700–0.814 |

*Blue=highest AUC (≥10% cutoff). AUC from roctab (Stata); 95% CI: asymptotic normal.*

**Supplementary Table 2: Agreement Matrix: PREVENT 3-tier vs Reti-CVD 3-tier (N = 1,224)**

| **PREVENT 3-tier** | **Reti-CVD Low  (n=549)** | **Reti-CVD Moderate  (n=512)** | **Reti-CVD High  (n=163)** | **Row Total** |
| --- | --- | --- | --- | --- |
| **Low (<5%) [n=1,003]** | 511 (50.9%) | 399 (39.8%) | 93 (9.3%) | 1,003 (81.9%) |
| **Moderate (5–10%) [n=150]** | 28 (18.7%) | 82 (54.7%) | 40 (26.7%) | 150 (12.3%) |
| **High (≥10%) [n=71]** | 10 (14.1%) | 31 (43.7%) | 30 (42.3%) | 71 (5.8%) |
| **Column total** | 549 (44.8%) | 512 (41.8%) | 163 (13.3%) | 1,224 |

*Yellow=PREVENT-Low reclassified Reti-CVD High. Blue=concordant High. κ unweighted=0.144  |  Quadratic Weighted κ=0.241  |  χ² P<0.001*

**Supplementary Table 3: Reclassification Table: PREVENT 4-tier vs Reti-CVD 3-tier (N = 1,224)**

| **PREVENT 4-tier (AHA)** | **Reti-CVD Low** | **Reti-CVD Moderate** | **Reti-CVD High** | **Row Total** |
| --- | --- | --- | --- | --- |
| **Low (<3%) [n=784]** | 427 (54.5%) | 300 (38.3%) | 57 (7.3%) | 784 (64.1%) |
| **Borderline (3–5%) [n=219]** | 84 (38.4%) | 99 (45.2%) | 36 (16.4%) | 219 (17.9%) |
| **Intermediate (5–7.5%) [n=93]** | 19 (20.4%) | 53 (57.0%) | 21 (22.6%) | 93 (7.6%) |
| **High (≥7.5%) [n=128]** | 19 (14.8%) | 60 (46.9%) | 49 (38.3%) | 128 (10.5%) |
| χ²(6)=154.2, P<0.001 |  |  |  |  |

*Yellow=PREVENT-Low reclassified Reti-CVD High. Blue=concordant High.*

*Among PREVENT <3%, 45.5% (357/784) were Reti-CVD Moderate or High.*
